# COGNITIVE PROFILES OF SPOUSAL AND PARENTAL CAREGIVERS: AN HRS STUDY

**DOI:** 10.1093/geroni/igac059.2071

**Published:** 2022-12-20

**Authors:** Katelyn Singer, Benjamin Katz

**Affiliations:** Virginia Tech Graduate School, Blacksburg, Virginia, United States; Virginia Tech, Blacksburg, Virginia, United States

## Abstract

Previous work analyzing the health and wellbeing correlates of caregiving has found increased rates of depression (Caputo, Pavalko, & Hardy, 2016), anxiety (Joling et al., 2015), and elevated risks of health problems, specifically cardiovascular-related incidents like hypertension (Capistrant, Moon, & Glymour, 2012), cardiovascular disease (Capistrant et al., 2012), and strokes (Haley et al., 2010). Research on specific cognitive effects that this responsibility may elicit has been less of an area of focus, especially when examining domains related to executive functioning. The present research investigated whether executive functioning mechanisms (such as processing speed, recall, and attention) are impacted in caregivers and if their function is mediated by interpersonal factors like birth year, gender, education, and depressive symptomology. Using the Health and Retirement Study, we compared spousal and parental caregivers of persons with medical conditions (n = 143) and non-caregivers (n = 975) who completed the 2015 Consumer and Activities Mail Survey (CAMS). Additional demographic and cognitive performance information was paired from the corresponding RAND 2016 data and the 2016 Harmonized Cognitive Assessment Protocol (HCAP) data. Preliminary ANCOVA results indicated, surprisingly, that spousal/parental caregivers were more likely to exhibit better executive function performance (p < .05) compared to non-caregivers, while delayed recall measures were not linked to caregiving status. These results indicate that intact executive function might be linked to one’s ability to provide caregiving for parents and/or spouses; while the HCAP was only administered at a single time point, future research should examine the longitudinal effects of caregiving on cognitive function.

